# Reaching consensus on definitions for food and physical activity policies: experience from the Policy Evaluation Network

**DOI:** 10.1093/eurpub/ckac147

**Published:** 2022-11-29

**Authors:** Liam Kelly, Cliona Twohig, Catherine B Woods, Aleksandra Luszczynska, Celine Murrin, Nanna Lien, Biljana Meshkovska, Carlijn B M Kamphuis, Maartje P Poelman, Laura Terragani, Sarah Forberger, Antje Hebestreit, Wolfgang Ahrens, Janas M Harrington

**Affiliations:** Department of Physical Education and Sport Sciences, Physical Activity for Health Research Cluster, Health Research Institute, University of Limerick, Limerick, Ireland; Centre for Health and Diet Research, School of Public Health, University College Cork, Cork, Ireland; Department of Physical Education and Sport Sciences, Physical Activity for Health Research Cluster, Health Research Institute, University of Limerick, Limerick, Ireland; Department of Psychology in Wroclaw, CARE-BEH Center for Applied Research on Health Behaviour and Health, SWPS University of Social Sciences and Humanities, Wroclaw, Poland; School of Public Health, Physiotherapy, and Sports Science, University College Dublin, Dublin, Ireland; Department of Nutrition, Faculty of Medicine, University of Oslo, Oslo, Norway; Department of Nutrition, Faculty of Medicine, University of Oslo, Oslo, Norway; Department of Interdisciplinary Social Science, Utrecht University, Utrecht, The Netherlands; Chair Group Consumption and Healthy Lifestyles, Wageningen University & Research, Wageningen, The Netherlands; Department of Nutrition, Faculty of Health Sciences, Oslo Metropolitan University (OsloMet), Oslo, Norway; Leibniz Institute for Prevention Research and Epidemiology—BIPS, Bremen, Germany; Leibniz Institute for Prevention Research and Epidemiology—BIPS, Bremen, Germany; Leibniz Institute for Prevention Research and Epidemiology—BIPS, Bremen, Germany; Centre for Health and Diet Research, School of Public Health, University College Cork, Cork, Ireland

## Abstract

**Background:**

An upsurge in policy evaluation research within public health sciences has led to multi-disciplinary research networks like the ‘Policy Evaluation Network’ (PEN). This multi-disciplinary collaboration highlighted the need for consensus on clear, common terminology and definitions to facilitate the multi-disciplinary research. This article outlines the development process of the PEN definitions glossary tool, with a focus on the key domains of policy design, implementation and outcomes as they apply to physical activity, sedentary behaviour and dietary behaviours.

**Methods:**

A project specific participatory process was undertaken, involving PEN researchers (*n* = 48) from seven European countries across various disciplinary backgrounds. All involved researchers were invited to identify and collate definitions that were commonly used in their research field. Terms and definitions were discussed and debated during three online workshops. Subsequently, the definitions were discussed and refined until consensus was reached.

**Results:**

Consensus definitions for 93 terms related to the evaluation of policy design, implementation and outcomes are provided. Consensus was reached on a range of terms where the terms were understood and used differently across represented disciplines (e.g. ‘Outcome’ and ‘Impact’). A conceptual ‘Inter-relations in policy-related concepts’ diagram was developed to enable navigation through an online database with key terms.

**Conclusions:**

The definitions resulting from this participatory process has supported PEN researchers and practitioners across disciplines to reach a shared understanding of different terms related to policy evaluation. Thus, providing a platform for avoiding conflicting use of the same terms in differing contexts over the course of the PEN work programme, facilitating clear and consistent communication, and allowing for clarity within collaborative multi-disciplinary projects and in public-facing messages.

## Introduction

As part of the Joint Programming Initiative on a Healthy Diet for a Healthy Life, researchers from 28 institutes in seven European countries and New Zealand combined their expertise to establish a multi-disciplinary research network—the Policy Evaluation Network (PEN). The network consists of a multi-disciplinary consortium with a vision to provide Europe with tools to identify, evaluate and benchmark policies designed to directly, or indirectly, address physical inactivity, unhealthy diets and sedentary behaviour (SB) while accounting for existing health inequalities.^1^ Members in the network span from a range of disciplines including nutrition, physical activity (PA), SB, health promotion, surveillance, health economics, epidemiology, health psychology and political science. The rationale for this glossary of definitions is aligned to the work of PEN,^1^ which aims to develop a consolidated approach to policy evaluation across Europe.

Multi-disciplinary research’ draws on knowledge from different disciplines,^2^^,^^3^ while multi-disciplinary research has been defined by the Organisation for Economic Co-operation and Development as the ‘interaction between two or more disciplines’.^4^ These interactions can range from communication of ideas to the mutual integration of organizing concepts, methodology, procedures, epistemology, terminology, data and organization of research and education in a large field. The PEN and other multi-disciplinary research groups face a range of challenges including achieving effective communication between discipline-based experts. The standardization and common understanding of terminology and definitions within research areas, and/or groups, is not a new problem. There is a tradition of developing definitions and terms across health science^5^ and other health-related multi-disciplinary research groups.^6^^,^^7^ However, achieving such standardization and shared understanding is often difficult.^5^ The lack of standardization and understanding is further compounded by the influence of external determinants and the commercial stakeholders or determinants in shaping policy development and policy definitions.^8^ Within the multi-disciplinary context of PEN, it quickly became evident that there was a need for the development of clear, common and accepted terminology and definitions. There was a lack of consistency amongst the terms used to describe similar concepts by partners with expertise in different disciplines (e.g. sport science, health promotion, surveillance scientists, health economists, epidemiologists, health psychology and political science researchers) across the PEN work packages (WP). But the challenge went even deeper. Based on the disciplinary location of the experts, the same terms were used for different concepts. For example, it was found that core terms, such as ‘Policy’ or ‘Policy Action’, were used in a variety of different contexts and the meaning inferred differed depending on the WP and discipline. A critical example of this was the understanding of the terms ‘impact’ and ‘outcome’, which varied between disciplines resulting in confusion particularly in relation to the time factor e.g. long-term outcome vs. short-term impact and vice versa. Early PEN publications also had conflicting definitions, highlighting the need to clarify terms and definitions before the project advanced further. For example, the PEN signature paper by Lakerveld et al.^1^ defines policies as ‘decisions, plans and actions that are enforced by national or regional governments which may directly or indirectly achieve specific health goals within a society’ (Page 2) whereas Zähringer et al.’s^9^ scoping review of nutrition and PA policies define policies as ‘decisions, plans, and actions that are undertaken to achieve specific health care goals within a society’ (Page 3). The PEN aims to go beyond the general approach of policy and specifically addresses public policy that is a form of government action usually expressed in a law, a regulation or an order. Since it reflects an intent of government or its representative entities.

While there are existing glossaries related to health promotion, i.e. the World Health Organization’s (WHO) ‘Health Promotion Glossary of Terms 2021’,^10^ to the authors’ knowledge, presently there is no standard set of definitions or terminology to be used within the context of PA, SB and healthy diet ‘policy design, implementation, evaluation, and key constructs of policy development’. We are also not aware that a glossary exists which explicitly addresses the nexus between public health research and political science and advances the understanding of shared terminology. Therefore, the methodology and results of studies are often difficult to interpret and compare. Cross-comparison with the WHO’s ‘Health Promotion Glossary of Terms 2021’ found little reference to policy-related definitions or terminology within the context of PA, SB and healthy diet. Thus, uncertainty in the use of terms amongst PEN partners persisted.

A PEN Glossary tool was not an original deliverable of the project, the development emerged as an informal process from an identified need. The design and development of the PEN Glossary are outlined here, however, a traditional ‘research design’ for the development of the Glossary was not employed due to the informal nature through which this need emerged. Further, a detailed evaluation of the Glossary was beyond the remit of the project. The Glossary as a tool met a project-specific need, i.e. an agreed understanding and agreed consensus on the use of terms across project WPs was essential for the success of PEN. This article outlines the PEN glossary and the ‘organic, collaborative and iterative’ process from which this Glossary was developed by PEN partners across all WPs. In that regard, this article should not be viewed as a product of research, but as an article presenting a process of scientific exchange and consensus aiming at enhancing cross-disciplinary understanding, and facilitating and enriching the communication between scientists, policy makers and multipliers. The aim of this article is to (i) outline the process used in the development of the PEN definitions glossary and (ii) present the set of definitions as agreed by the PEN partners.

## Methods

**Figure 1:**
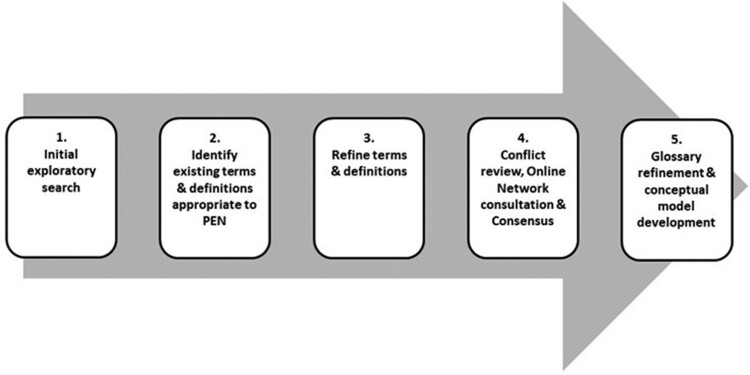
Process for developing PEN glossary of definitions and terms

Principles of ‘Participatory Action Research’ (PAR)^11^ were used as the adapted methodology for this research. PAR is based on data collection, reflection and action through engaging partners who are involved in the system, or project,^12^ i.e. PEN. The PAR process combines input across a variety of sectors, or experts, to enable the generation of new knowledge. This open-ended design, based on the principles of PAR, meant that the tool development evolved to the needs of the project as each step was completed. A PEN working group (Supplementary table S2a), led by partners in PEN WP1 (J.M.H., C.W., L.K. and C.T.), was established to identify existing glossaries of policy terms and definitions related to PA and healthy diets, and SB, and to compile a list of standardized definitions that could be incorporated throughout the PEN project. To reach a consensus on the definitions and terms to be used during PEN a modified approach similar to the ‘Nominal Group Approach’^13^ was used. This five-step process is outlined in figure 1.

### Step 1: initial exploratory search

All PEN members across each WP were contacted via the WP lead and asked to identify if they were aware of any existing glossary or compilation of standardized terms and definitions. The WP leads were asked to compile all WP responses and return to the Glossary subgroup (J.M.H., C.W., L.K. and C.T.). A subsequent exploratory search was completed to identify any existing glossary of policy-related terms specific to research in the fields of PA, SB, healthy diet, policy, evaluation, implementation and key constructs of policy development. Google Scholar and PubMed, as well as the official websites of international agencies tasked with public policy development, evaluation, or implementation, such as the Centre for Disease Control (CDC) POLARIS database, and the European Commission, were searched to identify any academic publications describing the development of a glossary of terms in the specified policy-related fields.

### Step 2: identify existing terms and definitions appropriate to PEN

Following identification and exploration of any existing glossary/data bases each of the PEN WPs (Supplementary table S1) were tasked with identifying definitions that they were using to operationalize their PEN research. An Excel workbook was shared with each WP. All partners were asked to identify definitions used in their specific tasks. Each WP lead was asked to compile their WP-specific terms and were instructed to enter the term, definition and source of the definition into a shared excel workbook. Definitions were then cross referenced between WPs and amalgamated where appropriate. Conflicts (where the same terms were used with different definitions), and variation of definitions were noted and brought forward to Step 3.

### Step 3: refine definitions and terms

Where varying definitions were submitted for the same term, the working group (Supplementary table S2a) either refined and agreed consensus on the definition most suitable to the needs of the project or developed one more suitable based on the information available. Definitions and terms were deemed inappropriate for refinement for the following reasons.

- There were multiple, contradictory definitions submitted for the same term (e.g. outcome referring to short-terms effects by one WP and/or discipline while being referred to as long-terms effects by other WPs and/or disciplines).
- The working group was not sure how to best refine the definitions submitted.

Once terms were refined these were again circulated to partners for another review. Partners were specifically asked to review the definitions used in their WP, and where these terms overlapped with other WPs.

### Step 4: conflict review/consensus, via online network consultation

An iterative approach with PEN partners and WP leads was adopted to resolve any conflicts and reach consensus. All partners and WP leads were invited to attend a series of three online workshops. The structured workshops were organized between July and September 2020. Each workshop lasted between 120 and 180 min and focused on the discussion of key terms where there was conflict, or multiple definitions, with a view to reaching consensus to ensure consistent use of terminology across all WPs within PEN. Each workshop was facilitated by a member of the PEN Glossary Working Group (*n* = 7; JH, CT, LK, CW, SF, AL and NL) and detailed notes of the discussions were taken. Breakout rooms were facilitated where necessary. Each workshop was also recorded. In total, 48 PEN members attended the workshops (Supplementary table S2b), with 11 PEN members representing all WPs attending all three workshops, each WP with its own scientific concept.

Defined objectives for all workshops included: (i) a review of current definitions as submitted by PEN partners, (ii) reflect on these terms and definitions with a view to discussing discrepancies and omissions, in smaller groups, (iii) provide feedback to main group to reach consensus, (iv) identify any missing terms and (v) finalize a list of definitions and terms to be sent to all PEN members after the session.

All PEN partners were then invited to review and provide feedback on any definitions where refinement was not possible. Input from all PEN WP partners was amalgamated (by C.T. and L.K.). Following a review of the definitions, the key thematic areas for the definitions were identified and revisions to the definitions, caveats, examples, and references were made.

### Step 5: Glossary refinement and conceptual model development

Based on workshop recordings and notes a final review and refinement of the definitions was completed. The draft glossary, including a visual glossary framework, was sent to all PEN partners for additional review and comments. After additional revisions, a revised draft of the glossary was resent to all members for comments, and group consensus. Consensus definitions were agreed by the Glossary working group, and the further revised glossary was recirculated to the PEN members for final comments. The final Glossary was presented to PEN partners and the PEN Scientific Advisory Panel during the PEN Mid-Term Symposium in November 2021 for final sign-off and approval.

## Results

Following Step 1, the CDC POLARIS database^14^ was identified, and many (but not all) of the PEN glossary terms submitted by PEN partners are referenced from this source. The CDC POLARIS database is a portal for navigating policy-relevant tools, trainings and resources. However, POLARIS alone did not comprehensively address all the terms and definitions being used in PEN. As outlined previously, some terms were submitted by more than one WP with differing or conflicting definitions depending on the discipline/WP from which they were submitted. Initially, definitions submitted by partners were grouped per WP, culminating in 93 definitions (Step 2). In 20 cases out of the 93 definitions identified, refinement was not appropriate (Step 3). These definitions (*n* = 20) would form the basis of the three workshops (Step 4) and are noted below. The remaining 73 definitions were approved for inclusion in the PEN Glossary.

### Workshop 1 (n=26 participants)

The definitions reviewed in Workshop 1 included: Policy, Policy Action, Policy Intervention Domain, Level, Area, Intervention, Programme and Environment. In Workshop 1, four questions were posed to participants:

1. What is understood by the PEN definition for policy?
2. What is the relationship between policy and intervention?
3. What is understood by the PEN definition for policy domains?
4. What is understood by the PEN definition for policy actions?

### Workshop 2 (n=27 participants)

Workshop 2 consisted of three parts. Part 1 presented the agreed definitions following Workshop 1 (Policy, Policy Action, Policy Intervention Domain, Level, Area, Intervention, Programme and Environment). Part 2 reviewed definitions for Policy Evaluation, Impact and Implementation. While Part 3 discussed Inter-related understandings. The four questions posed to participants in Workshop 2 were as follows:

1. What is understood by the PEN definition for policy implementation?
2. What is the relationship between policy implementation, process and evaluation?
3. What is understood by the PEN definition for policy evaluation?
4. What is the relationship between evaluation and impact?

### Workshop 3 (n=23 participants)

The definitions reviewed as part of Workshop 3 included: Impact, Outcome, Policy Implementation Evaluation, Policy Implementation Process and Inter-related understandings. The specific questions posed in Workshop 3 were:

1. What is understood by the PEN definition for Policy Implementation Evaluation?
2. What is understood by PEN definition for Policy Implementation Process?

Following the three workshops, the complete glossary was refined and circulated for final review by PEN partners and the co-ordinating team (tables 1 and 2). The final glossary is available at the https://www.jpi-pen.eu/. In total, the glossary includes definitions for 93 terms and concepts. The final consensus definitions for key terms (Policy, Policy Action, Policy Area, Policy Domain, Policy Evaluation, Policy Goal, Policy Impact, Policy Impact Evaluation, Implementation, Benchmarking, Framework and Government) are provided in table 1.

**Table 1:** PEN conceptual ‘Inter-relations in policy-related concepts’ diagram definitions (*n* = 15)

Term	Definition(s)
Policy	Policies are purposeful decisions, plans and actions made by voluntary or authoritative actors in a system designed to create system-level change to directly or indirectly achieve specific societal goals. Within this definition, public policy is a form of government action usually expressed in a law, a regulation or an order. Since it reflects an intent of government or its representative entities.^1^^,^^27^^,^^28^
Area	Specific content areas for policy actions within specific settings e.g. physical education and labelling.^29^
Domain	Components of the political system and/or settings organized around substantive issues. Policy domains differ depending on the target health goal/behaviour i.e. food or physical activity.
	Policy domains include settings e.g. health, agricultural, industrial, trade, transport, education, urban planning, economic, research & innovation and environment. Within policy domains, the context needs to be considered, such as geographical, epidemiological, socio-cultural, socio-economic, ethical, legal, organization and funding.^30–32^
Level	Laws, state-, district- & school-level codes or regulations, or class-level rules.
Setting	Refers to the specific environmental characteristics in which the actions are put into practice, including physical location or other policies implemented in the same time frame.^33–35^
Policy action	Policy actions are defined as actual options selected by policymakers. Public policy actions are specific actions put into place by any level of government or associated agencies to achieve the public health objective. They may be written into broad strategies, action plans, official guidelines/notifications, calls to action, legislation or rules and regulations. A policy action may have its own exclusive policy document or may be part of a larger document.
	Policy action is synonymous with policy intervention.
	For example, mandatory physical education or implementing a sugar-sweetened beverage tax.^29^^,^^36^
Indicators	Indicators are specific and measurable characteristics of changes that demonstrate progress towards outcome or impact. Indicators may be observable or not observable.^15^^,^^37^
Policy instruments	Techniques or means through which public actors (e.g. national and EU government bodies, public agencies, etc.) attempt to attain their goals. Examples of policy instruments are fiscal policies (taxes, subsidies), food standards, labelling regulations, education measures, etc.^32^^,^^35^^,^^38^^,^^39^ Different from intervention instruments.
Policy implementation evaluation	Evaluation principles and methods are used to understand how the policy was translated into operational practice, and/or to identify the occurrence or variation of intended and unintended outcomes and impacts.
	Implementation evaluation may compare and monitor different components or intensities of implementation or can inform efforts to identify and implement policy solutions by providing information about short-term outcomes, long-term impacts, knowledge, awareness, support, barriers and facilitators, sustainability and other implementation outcomes (see Supplementary appendix S1 and Reference^40^ for detailed implementation outcome examples).
	Policy implementation evaluations should apply an equity focus i.e. investigate whether effects differ for population subgroups (e.g. socio-economic groups, ethnic groups).^32^^,^^35^^,^^39^^,^^41^
Implementation outcomes	Under implementation evaluation, implementation outcomes are the effects of deliberate and purposive actions to implement new treatments, practices and/or services (for a list of examples see Reference ^40^ and Supplementary appendix S1).^40^
Policy implementation	An iterative process of policy making in which policy decisions are translated into practice.^32^^,^^35^^,^^39^
Policy implementation process	Policy implementation is an iterative process of policy making in which policy decisions are translated into practice (Ramesh and Perl, 2009; Howlet et al., 2009). Implementation process is realized by means of implementation strategies or instruments (Pfadenhauer et al., 2019). Policy implementation process is shaped by top-down actions, involving governments (their decisions and administrative practices), non-government (corporate) power structures and/or by bottom-up actions, involving stakeholders, and/or actors involved in the actual policy delivery (DeGroff and Cargo, 2009). Implementation process interacts with the characteristics of implementation setting or implementation context (values, culture, social, economic and political factors, etc.) (Pfadenhauer et al., 2019).^31^^,^^32^
Policy evaluation	The systematic collection or analysis of information to make judgments about contexts, activities, characteristics, outcomes (short-term) or impact (long-term) of one or more components of the policy process. Evaluation may inform or improve policy development, adoption, implementation or effectiveness, and may build the evidence base for policy actions/interventions.^9^^,^^41^
Outcome evaluation	Assesses the short-term, immediate effects of the intervention.^42^
Policy impact evaluation	Within a policy impact evaluation, evaluation principles and/or methods are used to examine long-term changes in key indicators that have occurred since the implementation of a policy, and/or the extent to which changes can be attributed to the policy. Policy impact evaluations should include unintended outcomes outside of the key indicators identified.
	Policy impact evaluations should apply an equity focus, and thus investigate whether effects differ for population subgroups (e.g. socio-economic groups, ethnic groups).^32^^,^^35^^,^^39^

**Table 2:** Additional glossary of definitions specific to PEN (*n* = 78)

Term	Definition(s)
Attribution	The extent to which the observed change in outcome is the result of the intervention, having allowed for all other factors which may affect the outcome(s) of interest.^23^
Benchmarking	Benchmarking is defined as the process of monitoring the performance of a country/city with respect to health policies and/or comparing this performance to an identified standard. Benchmarking can be done against a set of indicators or benchmarks identifying ‘best policy statements’ and/or against good practice examples of other countries.^43^
Benchmarks	Benchmarks or ‘good practice exemplars’ are the tools through which health promoting environments are created and/or assessed. They are comprehensive examples of policy implementation worldwide and are chosen based on their strength (e.g. external validated measures, such as using independent nutrient profiling criteria) and/or comprehensiveness (e.g. including a broad range of age groups, food groups, physical activity measures, media, settings or regions).^43^
Beneficiary	Beneficiaries are the individuals, firms, facilities, villages or similar that are exposed to an intervention with beneficial intentions. Synonymous with target group.^23^
Bias	The extent to which the estimate of impact differs from the true value as result of problems in the evaluation or sample design.^23^
Components	Components may refer to intervention components (e.g. multi-intervention components) OR environment policy index (EPI) components (e.g. classification of policy domains and policy areas).^43^
Conceptual systems models	For each case study in PEN work package 6, a specific conceptual systems model will be developed (using existing models as a starting point) that visualizes via which potential links, feedback loops and other dynamics the introduction of the policy could impact on (inequities in) diet and/or physical activity, and health in general.
	Such a conceptual systems model also visualizes how specific contextual factors play a role (related to the geographical, epidemiological, socio-cultural, socio-economic, ethical, legal and political context in which the policy is implemented), as well as specific characteristics of the setting.^32^^,^^35^^,^^39^
Confounding factors	Factors (variables) other than the intervention or policy which affect the outcome of interest.^23^
Context	Set of circumstances or unique factors that surround a particular implementation effort.^34^ To better address implementation challenges in different settings, it is important to understand what happens when evidence-based practice (e.g. intervention) is ‘woven together’ with a team, department or organization. In literature, ‘context’, ‘setting’ and ‘environment’ are often used interchangeably.^44^
Counterfactual	The state of the world in the absence of the intervention. For most impact evaluations, the counterfactual is the value of the outcome for the treatment group in the absence of the intervention. However, studies should also pay attention to unintended outcomes, including effects on non-beneficiaries.^44^
Data inventory	A data inventory includes metadata at different levels of aggregation. These datasets will be used to understand how indicators were operationalized across European countries and/or to document which monitoring/surveillance data are available for policy evaluation.^15^
Determinant	Factors believed or empirically shown to have a causal effect on implementation processes and/or implementation outcomes which may be targeted in order to achieve desired changes in individual, setting or system. This may include barriers (or hinders, impediments), facilitators (enablers) or other contextual determinants.^44^
Determinant framework	See framework.
Dimension	Indicator dimensions are determinants of diet/physical activity/sedentary behaviours and/or aspects of other unobservable determinants set within a domain.
Diversity	Understanding and recognizing individual differences, in terms of race, ethnicity, gender, sexual orientation, socio-economic status, age, physical abilities, religious beliefs, political beliefs or other ideologies.^45^
Domains	See policy domain.
Ecological environment	Nested arrangement of concentric structures, each contained within the next, referred to as the micro-, meso-, exo- and macro-systems.^33^
Encouragement design	A form of randomized control trial in which the treatment group is given an intervention (e.g. a financial incentive or information) to encourage them to participate in the intervention being evaluated. The population in both treatment and control has access to the intervention being evaluated, so the design is suitable for national-level policies and programmes.^23^
Environment	The collective physical, economic, policy and socio-cultural surrounding, opportunities and conditions that influence people’s lifestyle choices and behaviours for the prevention of NCDs.
Equity	See health equity.
Ethnic minority	An ethnic minority is a group of people who differ in race or in national, religious or cultural origin from the dominant group—often the majority population—of the country in which they live.^46^
Evaluation framework	See framework.
Evidence (Food-EPI)	For each of the Food-EPI indicators evidence of implementation by the government will be collected in each country and for the EU, by comprehensively reviewing policy documents of committee reports in each of the countries. This information will be used to ground good practice indicators in the available evidence in each country, e.g. to determine the existence and degree of implementation of a particular policy.^43^
Ex ante evaluation design	An impact evaluation design prepared before the intervention takes place. Ex Ante Designs are stronger than ex post-evaluation designs because of the possibility of considering random assignment, and the collection of baseline data from both treatment and comparison groups. Also called prospective evaluation.^23^
Ex post-evaluation design	An impact evaluation design prepared once the intervention has started, or possibly been completed. Unless there was random assignment then a quasi-experimental design must be used.^23^
Exo-system	One or more settings that do not involve the developing person as an active participant, but in which events occur that affect, or are affected by, what happens in the setting containing the developing person.^33^
Experimental (randomized) evaluations	An impact evaluation design in which random assignment has been used to allocate the intervention amongst members of the eligible population. Since there should be no correlation between participant characteristics and the outcome, and differences in outcome between the treatment and control can be fully attributed to the intervention, i.e. there is no selection bias. However, EDs may be subject to several types of bias and so need follow strict protocols.^23^
Food	Refers to food and non-alcoholic beverages. It excludes breastmilk or breastmilk substitutes.^47^
Food-EPI	The Healthy Food Environment Policy Index (Food-EPI) is a monitoring tool to assess government policies and actions for creating healthy food environments against international best practice.
Food poverty	Food poverty is the inability of individuals and households to obtain an adequate and nutritious diet, often because they cannot afford healthy food or there is a lack of shops in their area that are easy to reach.
	Synonymous with food insecurity.^48^
Framework	Frameworks are defined as a graphical or narrative representation of the key factors, concepts or variables to explain the phenomenon under study, and as a minimum need to include the steps, strategies or factors relevant for the various stages of the development.
	Frameworks may include evaluation implementation frameworks, determinant frameworks or conceptual frameworks.^49–51^
Good practice exemplars	See benchmarks.
Government	Includes any government departments or, where appropriate, other agencies (i.e. statutory bodies such as offices, commissions, authorities, boards, councils, etc.).^47^
Guidelines	The processes of health policymaking. This may include guidelines in specific setting as part of policy implementations—i.e. in hospitals or related to school meals.
	Guidelines are formal advisory statements containing recommendations, which tells the intended end-user of the guideline what he or she can or should do in specific situations to achieve the best health outcomes possible, individually, or collectively. They should be robust enough to meet the unique circumstances and constraints of the specific situation to which they are being applied.^52^
Harmonization	Harmonization refers to the process of minimizing differences in comparability of measures, variables and/or methods, so that data are comparable across surveys, age groups and/or countries. This will include an agreed set or suite of indicators, which provide comparable data for evaluation across existing surveillance systems.^53^
Health equity	The absence of avoidable, unfair or remediable differences among groups of people, whether those groups are defined socially, economically, demographically or geographically or by other means of stratification.^54^
Healthy/unhealthy food	Categorization of foods as healthy/unhealthy is determined according to extent to which a diet pattern consisting of these foods may protect against malnutrition in all its forms, as well as non-communicable diseases (NCDs).
	Where it is not clear which category to use, categorization of foods should be informed by rigorous criteria or the use of a nutrient profiling model.^47^
Implementation complexity	Perceived difficulty of implementation, reflected by duration, scope, radicalness, disruptiveness, centrality and/or number of steps required to implement.^34^
Implementation frameworks	See framework.
Implementation quality	Consists of two components: the intervention itself, and the support system. Measurement of implementation quality of both the intervention and support system should include assessments of adherence in terms of fidelity (degree to which an intervention and its support system are conducted as planned), dosage (specific units of an intervention and support system) and/or quality of delivery (affective engagement, sensitivity and responsiveness).^55^
	Quality of delivery is related to quality of process, a term used to highlight the importance of engaging participants in an intervention or the reciprocal nature of interactions that are necessary for learning and behaviour change.^55^^,^^56^
Implementation science	Scientific study of methods to promote the systematic uptake of research findings and/or other evidence-based practices into routine practice, and hence, to improve the quality and effectiveness of health services. It includes the study of influences on healthcare professional and/or organizational behaviour.^57^
Intervention	An umbrella term, which includes any policy, programme or environmental change (physical and/or social) used to promote specific health behaviours or goals. Different from policy action/policy intervention.^29^
Intervention instruments	Instruments are methods or tools that measure variables to assess the key indicators necessary for policy evaluation. Suitable instruments to assess selected variables of key indicators (or the indicators directly) have to be valid and reliable; should overlap with different existing systems; and must be easily applicable to provide robust estimates.^15^ Different to policy instruments.
Intervention impact evaluation	Assesses the long-term effects of the intervention on participants and other stakeholders. Baseline data at the start of the programme are compared to data collected at follow up time points during service delivery.^42^
Intervention implementation	Act of converting programme objectives into actions through deployment of resources, policy changes, regulations including the coordination or supervision of activities in support of the planned interventions.^58^
Macro-system	Consistencies, in the form and content of lower-order systems (micro-, meso-, exo-) that exist, or could exist, at the level of the subculture or the culture as a whole, along with any belief systems or ideology underlying such consistencies.^33^
Meso-system	Inter-relations among two or more settings in which the developing person actively participates (such as, for a child, the relations among home, school or neighbourhood peer group; for an adult, among family, work or social life).^33^
Micro-system	A pattern of activities, roles or interpersonal relations experienced by the developing person in a given setting with particular physical or material characteristics.^33^
Model	Theories with a more narrowly defined scope of explanation; a model is descriptive, whereas a theory is explanatory as well as descriptive.^50^
Monitoring	Monitoring is the continuous, systematic collection, analysis and interpretation of health-related data on specified indicators needed for the planning, implementation or evaluation of policies. It can include the integration of a multitude of types of evidence, both qualitative and/or quantitative, to provide the main stakeholders with indications of the extent of progress and achievement of objectives or progress in the use of allocated funds and intervention development. Synonymous with surveillance.^15^^,^^59^^,^^60^
Non-communicable diseases	Non-communicable diseases (NCDs), also known as chronic diseases, tend to be of long duration and are the result of a combination of genetic, physiological, environmental or behaviours factors.^61^
Nutrient profiling model	Nutrient profiling is a tool used to categorize foods and non-alcoholic beverages according to those that are more likely to be part of a healthy diet from those that are less likely. This is often based on foods which contribute to excess consumption of energy, saturated fats, trans fats, sugar or salt.^62^
Policy goals	Goals are statements that describe the fundamental outcomes that a policy aims to achieve through its activities. Policy goals are high order statements of desired outcomes (e.g. reduced environmental impact). Outcomes are often divided in short, intermediary and long-term, where the latter often is called impact (see policy impact definition).^63^
Policy impact	Refers to all possible economic, social, political, technical and ecological effects at local, regional or national level that have a direct or indirect effect on the target group or other parties. It includes all significant longer-term effects directly or indirectly, intended or unintended, on the ultimate stakeholders and third parties.^15^^,^^41^^,^^63^
Policy implementation variables	Determinants, barriers and facilitators, defined as variables that are hypothesized or have been found to influence implementation processes or implementation outcomes. In policy implementation research, strategies to facilitate implementation are referred to as policy instruments (or government instruments).^50^
Policy intervention	Synonymous with policy action.
Policy level	These are the level in the system where policies can be targeted. This includes laws, state-, district- & school-level codes or regulations, or class-level rules.
Policy outcome	Policy outcomes are short-term and intermediate changes in target organizational, societal, or cultural norms, i.e. audience behaviour, awareness, attitudes or knowledge. Desired outcomes are termed policy objectives.^15^^,^^41^^,^^64^
Policy outcome evaluation quality	Quality policy outcome evaluation requires selection of a robust and well-suited methodology, which should explore counterfactuals, quantify impacts across different levels of policy implementation or different population groups, study both direct and indirect effects, control for confounding factors and self-selection, and ideally be replicable by third parties.^65^
Policy output	Direct products or deliverables that result from activities. Outputs generally are observed immediately and do consist of a formal evaluation component.^41^
Political actors/stakeholders	Political decision makers of different policy levels: city/local, regional/county, state, national and International. Examples will differ depending on country/context.^66^^,^^67^
Programme	This is a type of intervention. Time-limited opportunities for the purpose of increasing targeted health behaviours, attitudes or knowledge in a target population.^29^
Public policy implementation	Refers to the transformation of government decisions through processes including different levels of government, administrative structures and capacities, inner administrative dynamics, party interest and/or underlying normative and power structures.^31^^,^^68^^,^^69^
Quality implementation	Putting an innovation into practice in a way that meets the necessary standards to achieve the innovation’s desired outcomes.
	Relies on three theoretical assumptions about innovations: innovations need to be well defined and include specific standards for implementation. The process of putting an innovation into practice includes monitoring and evaluating activities. Innovations often need to be adapted or modified to fit the host setting within which they will be implemented.^70^
Quasi-experimental evaluations	Impact evaluation designs used to determine impact in the absence of a control group from an experimental design if the control group is not randomly assigned. Many quasi-experimental methods, e.g. propensity score matching and regression discontinuity design, create a comparison group using statistical procedures. The intention is to ensure that the characteristics of the treatment and comparison groups are identical in all respects, other than the intervention, as would be the case from an experimental design. Other regression-based approaches have an implicit counterfactual, controlling for selection bias and other confounding factors through statistical procedures.^23^
Screeners	Screeners are short screening instruments which consist of a set of simple standard instruments/questions measuring variables that are needed to describe the most relevant indicators in the full surveillance sample. Further, they should be self-sufficient for inclusion in different survey instruments to be used in calibrating existing instruments with which they overlap across different surveillance systems. Their selection for specific topics should be based on validity, reliability and the evidence regarding impact on health and health behaviour.^53^
Sedentary behaviour	Sedentary behaviour is any waking behaviour characterized by an energy expenditure ≤1.5 metabolic equivalents (METs), while in a sitting, reclining, or lying posture.^5^
Setting	Refers to the specific environmental characteristics in which the actions are put into practice, including physical location or other policies implemented in the same time frame.^33–35^
Social or social-cultural actors/stakeholders	These include teachers, educators, students, public health researchers etc. Institutions associated with these stakeholders include universities, schools, kindergartens, physical activity clubs, consumer organizations or research groups external to PEN.
Socio-economic position	The social or economic factors that influence what positions individuals or groups hold within the structure of a society.^71^
Socio-political actors/stakeholders	Individual actors or representatives of organizations and associated with public health areas, such as food, physical activity or sports. This includes non-governmental organizations including national nutrition agencies, physical activity agencies, national research organizations and funding bodies, sports federations, business associations, voluntary and charitable organizations.
Sugar-sweetened beverages/sugar-sweetened drinks tax	Taxes on sugar-sweetened beverages (SSB) or sugar-sweetened drinks (SSD), which are not to contribute to excess consumption of the nutrients of concern, increasing the risk of NCD’s. Drinks may be taxed based on their classification (e.g. soda or bottled iced tea) OR based on the amount of added sugar and a total sugar content per unit volume.^72^
Stakeholders/actors	Stakeholder or actors are ‘individuals, organizations or communities that have a direct interest in the process or outcomes of a project, research or policy endeavour’. The types of stakeholders may vary and can include social or social-cultural, socio-political actors or political actors.^73^^,^^74^
SUMPs	Sustainable Urban Mobility Plans (SUMPs) aim to improve the accessibility of urban areas and to raise the attractiveness, safety and security of walking or cycling, in order to raise PA on a population level. SUMPs encourage active transport by improving or developing dedicated infrastructure for cyclists and pedestrians to separate them from heavily motorized traffic or by reducing travel distances. Infrastructural changes should be complemented by other technical, as well as policy-based, ‘soft’ measures.^75^
Surveillance	Synonymous with monitoring.
Systems model	A complex systems model of public health conceptualizes poor health and health inequalities as outcomes of a multitude of interdependent elements within a connected whole. Complex systems are defined by several properties, including emergence, feedback or adaptation.^35^
Target group	Synonymous with beneficiary.
Theory	Set of analytical principles or statements designed to structure our observation, understanding or explanation of the world.^50^

**Figure 2:**
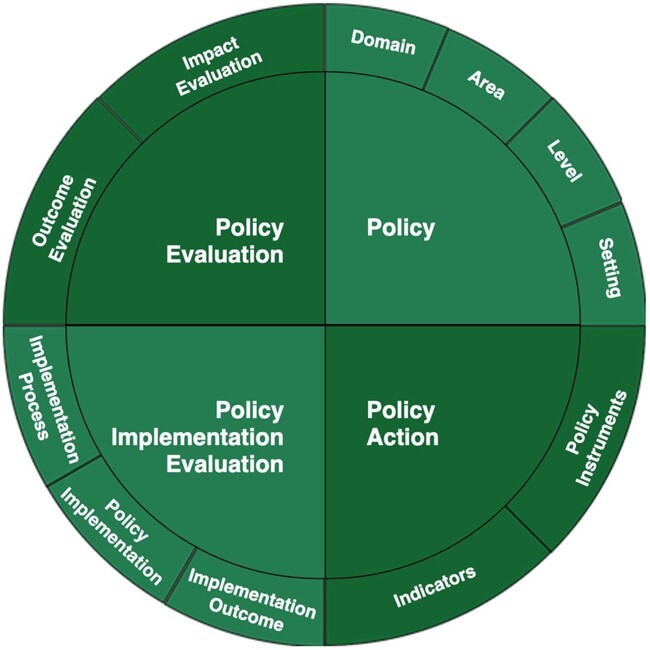
Conceptual ‘Inter-relations in policy-related concepts’ diagram specific to PEN

A conceptual ‘Inter-relations in policy-related concepts’ framework (the glossary framework) was developed to enable navigation through the key terms, to illustrate how the various terms and definitions used across PEN fit together (figure 2). This interactive tool is available on the PEN website (https://www.jpi-pen.eu/pen-glossary-of-definitions.html).

Table 2 provides additional PEN definitions included in the Glossary.

## Discussion

The aim of this article was to describe the process of reaching a consensus for definitions of terms relevant to policy evaluation research and to present a framework to illustrate the connections across key terms (figure 2) used in the PEN. The resultant glossary and interactive tool is open access and freely available to researchers (https://www.jpi-pen.eu/pen-glossary-of-definitions.html).

As noted, the prominence of policy evaluation research is continuing to expand.^15–18^ The experience of the PEN discussion on terminology and definitions highlighted the need for the development of clear, common and accepted terminology and definitions for the success of the project. This need was identified in the PEN and mirrors existing needs highlighted in other research.^5^^,^^19^ It is also likely to be a topic relevant to other multi-disciplinary research consortia and other researchers in this area. However, such standardization can be difficult to achieve across, and within, multi-disciplinary teams. There are some excellent resources available to locate and understand key terms in an efficient way. For example, the CDC POLARIS database (Office of the Associate Director for CDC Policy and Strategy, 2019), a portal for navigating policy-relevant tools, training, and resources. While this database provided the basis for many of the terms and definitions within the PEN Glossary,^14^ PEN identified an additional need for a consensus and standardization of definitions specifically in the context for healthy diet, SB and PA policy development, implementation and evaluation.

## Strengths and limitations

Many of the world’s contemporary challenges, including those related to PA, sedentary and dietary behaviours are inherently complex and cannot be addressed by any single discipline, requiring a multifaceted and integrated approach across disciplines. The benefits of inter- and multi-disciplinary research approaches have been documented.^20^^,^^21^ While PEN is a multi-disciplinary consortium by definition^22^ drawing on knowledge from different disciplines but staying within each disciplinary boundary, the development of the glossary could be described as taking a multi-disciplinary approach, which analyzed, synthesized and harmonized the links between disciplines to understand the differences in perspectives to reach a consensus on definitions (Choi et al.). Rather than working independently and using definitions from a disciplinary perspective, PEN researchers across disciplines worked interactively and collaboratively to collectively reach a consensus on definitions in the PEN Glossary.

Due to the ‘emergent’ nature and application of this glossary of terms there are a number of limitations to be considered in the context of how the consensus of definitions was reached. The Glossary tool was created within the context of PEN, a European research project, where the majority of the definitions suggested were from researchers within a European context. It should be acknowledged that inconsistency across terms and definitions in the policy field is not a new concept. Indeed, Birkland identifies multiple definitions of what constitutes public policy, and those public policies have several common characteristics (Birkland, 2015).

The Glossary, which developed organically, was not an intended original deliverable for PEN and the process could be described as being outside a ‘traditional research process’. In an ideal scenario without the time constraints of meeting proposed milestones and deliverables in a timely manner the first step would have been to conduct a systematic literature review of existing terms and definitions to help refine methods and to specify specific research questions. Definitions and terms, which frequently caused misunderstanding amongst project partners, were collected. Experts within the PEN consortium provided input to the consensus definitions, which is a clear strength and should help with adoption and acceptance of these definitions. The authors acknowledge that it would have been advantageous for the final set of definitions and terms to be validated by an independent external panel or group, but due to time constraints this was not feasible with the glossary primarily developed as a tool facilitating collaboration across the various disciplines involved in PEN, thus serving the needs of our network. The ‘Glossary’ development process was however presented to the Scientific Advisory Panel at the mid-term symposium. The proposed definitions may not always be completely suitable to the needs of other multi-disciplinary projects and therefore may require some adaptation, particularly if not related to PA, SB, or healthy diet or if to be used in a discipline outside the remit of PEN. However, there will likely be other definitions that exist for good reasons or that will emerge as research evolves. Additionally, some definitions sourced were interspersed in other learning resources (CDC) or in specific contexts (Impact Evaluation Glossary^23^). These definitions were often very specific, and not generic enough to apply to across all PEN WPs. The goal of this project was not to marginalize such opinions, but rather responds to calls for better standardization and harmonization of work in the field at this point in time. Similarly, the development of the glossary did not involve policymakers or other stakeholders responsible for implementation and evaluation of PA/healthy diet policies. Acknowledging the focus of PEN was specifically on public policies, the definition of policy adopted reflected this. However, the definition of policy adopted does not address the external extant tensions as a result of the commercial influence on actions (or non-actions) by Government actors.^24^ The glossary presented builds on previous work (i.e. existing definitions adopted to the needs of the PEN project) and may be continued as the multi-disciplinary field of ‘Policy Evaluation’ develops. Further steps could potentially include testing the validity of the proposed definitions in a broader stakeholder group.

For the purpose of PEN all disciplines needed were represented. However, we acknowledge that the contributions of researchers from a wider geographical span and from other disciplinary areas would be important for future development of the glossary. Notably, one of the key strengths of this study is that it developed ‘organically’ to address the needs of the PEN project and to address an important gap in healthy diet and PA policy research and evaluation.

The CDC logic model tool for planning, describing, managing, communicating and evaluating a programme or intervention^25^ was used by PEN as a point of departure framework. It recognizes the steps from identifying the problem relating to current policies towards design, implementation and outcomes. However, to achieve the aims of PEN and to carry out all research activities in the seven WPs in a coherent and consistent way, Kamphius et al.^26^ developed an overarching theory-based framework, which visualizes the interplay between policy domains (i.e. policy development, policy implementation and policy outcomes), acknowledging the complex and dynamic inter-relations between policy domains, the influence of contextual factors and the importance of equity considerations in all policy domains. The use of consistent terminology and definitions was essential for this process.

## Potential for a collaborative standardization of common terms

The glossary of definitions and terms was developed initially for use within the PEN project and PEN researchers were encouraged to consult the glossary as their work progressed and manuscripts were being written. For example, the working group which developed the PEN theoretical framework, Kamphuis et al.^26^ cross referenced the terms used in the development of the framework against those in the Glossary to ensure consistency. As new projects emerged, collaborations between four large European Funded consortia (PEN, STOP, Co-Create and Best ReMap) were established, given they all focus on policy development, implementation and evaluation for healthier lifestyles to address some of the major public health challenges of the 21st century. The glossary as a live tool has the potential to be further developed as these collaborations mature. The collaboration provides the opportunity to provide a comprehensive, transparent and sustainable glossary of standardized definitions and terms that potentially can be adopted across multiple projects, thereby advancing future research related to policy development, implementation and evaluation for healthier lifestyles. However, such an endeavour is not without its challenges and would depend on a project, or team, taking responsibility for maintaining the glossary and keeping it up to date.

## Conclusion

The definitions arrived at by the PEN consortium are presented as standardized definitions for use both within and beyond PEN as described. Periodic reviews of these consensus definitions were conducted throughout the duration of the PEN project, and updates made when appropriate. The Glossary is publicly available on the PEN website (https://www.jpi-pen.eu/). It is envisaged that the definitions resulting from this transparent, and broad-based participatory process will allow a greater understanding of shared terminology across and between disciplines. The challenge to maintain and update the glossary post-PEN is a real though, not an unsurmountable one. For example, previous work from the JPI DEDIPAC Knowledge Hub maintained the Determinants of Nutrition and Eating Framework (‘DONE’) interactive resource (https://www.uni-konstanz.de/DONE/) on conclusion of the project. This is a model, which could be explored for the maintenance of the PEN Glossary.

## Supplementary Material

ckac147_Supplementary_Data

## Data Availability

The data underlying this article are available in the article Availabilityand in its online supplementary material.
