# Supplementary material for: Reaching consensus on definitions for food and physical activity policies: experience from the Policy Evaluation Network
Source: Eur J Public Health. 2022 Nov 29;32(Suppl 4):iv10–20. doi: 10.1093/eurpub/ckac147 (PMC9706120; doi:10.1093/eurpub/ckac147)
Supplement: ckac147_Supplementary_Data [file ckac147_supplementary_data.docx]

**Supplementary Tables**

**Table 1. PEN Consortium Partners**

| # | **Organisation legal name** | **Acronym** | **PEN Group leader** | **City, Country** |
| --- | --- | --- | --- | --- |
| 1 | University College Cork | CHDR | Dr Janas Harrington | Cork, Ireland |
| 2 | Medical University of Silesia in Katowice | SILVeR | Prof Piotr Romaniuk | Katowice, Poland |
| 3 | University College Dublin | UCD-PH | Dr Celine Murrin | Dublin, Ireland |
| 4 | Vrije Universiteit | VU-P&P | Prof Ingrid Steenhuis | Amsterdam, The Netherlands |
| 5 | VU University Medical Center | VUmc | Dr Jeroen Lakerveld | Amsterdam, The Netherlands |
| 6 | Erasmus Medical Center Rotterdam | SocialEpiEMCR | Prof Frank van Lenthe | Rotterdam, The Netherlands |
| 7 | Oslo Metropolitan University | OsloMet | Dr Gun Roos | Oslo, Norway |
| 8 | University of Bologna | UNIBO | Prof Mario Mazzocchi | Bologna, Italy |
| 9 | Academic Medical Center, University of Amsterdam | PHP-DP | Prof Karien Stronks | Amsterdam, The Netherlands |
| 10 | Ludwig-Maximilians-Universität München | EBPH-LMU | Dr Eva Rehfuess | Munich, Germany |
| 11 | Helmholtz Zentrum München | T-HMGU | Dr Michael Laxy | Munich, Germany |
| 12 | Gdansk University of Technlogy | GUT | Prof Joanna Zukowska | Gdansk, Poland |
| 13 | Grenoble Applied Economics Laboratory, French National Institute of Agricultural Research | GAEL- INRA | Dr Laurent Muller | Grenoble, France |
| 14 | SWPS University of Social Sciences and Humanities | SWPS | Prof Aleksandra Luszczynska | Warsaw, Poland |
| 15 | Poznan University of Medical Sciences | Po-LI | Prof Katarzyna Wieczorkowska-Tobis | Poznan, Poland |
| 16 | German Cancer Research Center | DKFZ | Prof Hermann Brenner | Heidelberg, Germany |
| 17 | Università Cattolica del Sacro Cuore | UCSC | Prof Daniele Moro | Milan, Italy |
| 18 | University of Limerick | UL | Prof Catherine Woods | Limerick, Ireland |
| 19 | Medical Center – University of Freiburg | CG-IEM | Dr Joerg J. Meerpohl | Freiburg, Germany |
| 20 | Friedrich‐Alexander University, Institute of Sport Science and Sport | FAU-ISS | Prof Alfred Rütten | Erlangen, Germany |
| 21 | University of Oslo, Institute of Basic Medical Sciences, Department of Nutrition | UiO-PHN | Prof Nanna Lien | Oslo, Norway |
| 22 | University of Ulm, Division of Sport and Rehabilitation Medicine | UULM | Prof Juergen Steinacker | Ulm, Germany |
| 23 | Robert Koch Institute | RKI | Dr Gert Mensink | Berlin, Germany |
| 24 | Johannes Gutenberg University Mainz | JGU | Prof Thomas Kubiak | Mainz, Germany |
| 25 | Leibniz Institute for Prevention Research and Epidemiology - BIPS | BIPS | Prof Wolfgang Ahrens | Bremen, Germany |
| 26 | Alimentation et Sciences Sociales | INRA ALISS | Dr Olivier Allais | Ivry-Sur-Seine, France |
| 27 | Faculty of Geosciences, Utrecht University | HUL | Dr Carlijn Kamphuis | Utrecht, The Netherlands |
| 28 | University of Auckland | UOA | Dr Stefanie Vandevijvere | Auckland New Zealand |

**Table 2a. PEN Glossary Working Group**

| **Title** | **Name** | **Affiliation** | **Area of Expertise** |
| --- | --- | --- | --- |
| Prof | Catherine Woods | Physical Activity for Health Research Cluster, Health Research Institute, University of Limerick, Ireland. | Physical Activity Policy Evaluation and Development |
| Dr | Janas Harrington | Director BSc Public Health Science, School of Public Health, University College Cork, Ireland | Nutritional Epidemiology, Public Health Nutrition, Obesity |
| Dr | Liam Kelly | Physical Activity for Health Research Cluster, Health Research Institute, University of Limerick, Ireland. | Physical Activity & Health Policy |
| Dr | Cliona Twohig | HRB Centre for Health & Diet Research, School of Public Health, University College Cork, Ireland | Research Assistant & Registered Dietitian |
| Dr | Sarah Forberger | Leibniz-Institute for Prevention Research and Epidemiology – BIPS, Department Prevention and Evaluation, WHO Collaborating Centre for Obesity Prevention, Nutrition and Physical Activity | Public Policy Analysis |
| Prof | Nanna Lien | Faculty of Medicine, Institute of Basic Medical Sciences, Norway | Public Health Nutrition |

**Table 2b. PEN Glossary Workshop Attendees**

|  | Name | Work Package | | Workshop 1  (n=23) | | Workshop 2  (n=27) | | Workshop 3  (n=26) |
| --- | --- | --- | --- | --- | --- | --- | --- | --- |
| 1 | Marion Flechtner | WP3 / WP4 | | Y | | Y | |  |
| 2 | Lucia Reisch | WP6 | | Y | |  | |  |
| 3 | Kevin Volf | WP1 | | Y | | Y | | Y |
| 4 | Agnieszka Neumann-Podczaska | WP1 | | Y | | Y | | Y |
| 5 | Nanna Lien | WP2 / WP3 / WP4 | | Y | | Y | | Y |
| 6 | Biljana Meshkovska | WP6 | | Y | | Y | | Y |
| 7 | Wolfgang Ahrens | WP7 | | Y | | Y | | Y |
| 8 | Marie Scheidmeir | WP4 / WP5 | | Y | | Y | |  |
| 9 | Tobias Niedermaier | WP3 | | Y | |  | |  |
| 10 | Beatrice Biondi | WP3 | | Y | | Y | | Y |
| 11 | Antje Hebestreit | WP2 | | Y | |  | |  |
| 12 | Gun Roos | WP5 | | Y | |  | |  |
| 13 | Renee Stark | WP3 | | Y | | Y | |  |
| 14 | Karolina Lobczowska | WP4 / WP5 | | Y | | Y | |  |
| 15 | Stefanie Do | WP2 | | Y | |  | | Y |
| 16 | Joerg J. Meerpohl | WP6 | | Y | |  | |  |
| 17 | Enrique Garcia | WP1 | | Y | | Y | | Y |
| 18 | Laura Terragn | WP5 | | Y | | Y | |  |
| 19 | Liam Kelly | WP1 / WP7 | | Y | | Y | | Y |
| 20 | Cliona Twohig | WP1 | | Y | | Y | | Y |
| 21 | Janas Harrington | WP1 | | Y | | Y | | Y |
| 22 | Catherine woods | WP1 / WP7 | | Y | | Y | | Y |
| 23 | Aleksandra Luszczynska | WP4 / WP5 | | Y | |  | |  |
| 24 | Celine Murrin | WP2 | |  | | Y | |  |
| 25 | Jeroen Lakerveld | WP1 | |  | | Y | |  |
| 26 | Karien Stronks | WP5 | |  | | Y | |  |
| 27 | Nicole Den Braver | WP1 | |  | | Y | |  |
| 28 | Joanna Zukowska | WP1 | |  | | Y | |  |
| 29 | Laurent Mueller | WP3 | |  | | Y | |  |
| 30 | Sigrun Henjum | WP5 | |  | | Y | |  |
| 31 | Hajo Zeeb | WP4 | |  | | Y | |  |
| 32 | Janine Wendt | WP4 / WP6 | |  | | Y | |  |
| 33 | Anne Lene Løvhaug | WP5 | |  | | Y | |  |
| 34 | Sarah Forberger | WP1 | |  | | Y | | Y |
| 35 | Romanika Okraszewska | WP1 | |  | |  | | Y |
| 36 | Ina Alvarez | WP7 | |  | |  | | Y |
| 37 | Carlijn Kamphuis | WP5 / WP6 / WP7 | |  | |  | | Y |
| 38 | Clarissa Leydon | WP1 | |  | |  | | Y |
| 39 | Sven Messing | WP1 / WP2 | |  | |  | | Y |
| 40 | Tobias Niedermaier | WP3 | |  | |  | | Y |
| 41 | Maartje Poelman | WP2 | |  | |  | | Y |
| 42 | Isobel Stanley | WP2 / WP3 |  | |  | | Y | |
| 43 | Thomas Kubiak | WP4 |  | |  | | Y | |
| 44 | Sanne Djojosoeparto | WP1 |  | |  | | Y | |
| 45 | Katarzyna Brukało | WP1 / WP4 |  | |  | | Y | |
| 46 | Krzysztof Kaczmarek | WP4 |  | |  | | Y | |
| 47 | Lina Garnica Rosas | WP2 |  | |  | | Y | |
| 48 | Wolfgang Ahrens | WP7 | Y | | Y | | Y | |

Key: WP = Work Package; WP1 - Policy mapping and EPI development; WP2 - Monitoring and surveillance; WP3 - Estimation and simulation of policy impact; WP4 - Policy implementation evaluation; WP5 - Equity and diversity of policies; WP6 - Policy in practice - Selected case studies; WP7 - Network coordination and dissemination.
